# Use of Propolis in the Sanitization of Lettuce

**DOI:** 10.3390/ijms150712243

**Published:** 2014-07-09

**Authors:** Xesús Feás, Lazaro Pacheco, Antonio Iglesias, Leticia M. Estevinho

**Affiliations:** 1 Department of Organic Chemistry, Faculty of Sciences, University of Santiago de Compostela, E-27080 Lugo, Spain; E-Mail: xesus.feas@usc.es; 2 CIMO-Mountain Research Center, Agricultural College of Bragança, Polytechnic Institute of Bragança, Campus Santa Apolónia, E 5301-855 Bragança, Portugal; E-Mail: pacheco@hotmail.com; 3 Department of Anatomy and Animal Production, Faculty of Veterinary Science, University of Santiago de Compostela, E-27002 Lugo, Spain; E-Mail: antonio.iglesias@usc.es

**Keywords:** lettuce, propolis, sanitization, microbiological safety, sodium hypochlorite

## Abstract

The present study aimed to determine the effectiveness of propolis in reducing the microbial load in ready-to-eat (RTE) and fresh whole head (FWH) lettuces (*Lactuca sativa* L.) type Batavia. Two sanitizing solutions were employed: sodium hypochlorite (SH) and propolis (PS), during 15 and 30 min. Tap water (TW) was used as a control. Regarding the mean reduction on aerobic mesophiles, psychrotrophic and fecal coliforms, the SH and PS treatments showed the same pattern of variation. In all cases, PS was slightly more effective in the microbiological reduction in comparison with commercial SH. Reductions between two and three log cycles were obtained with PS on aerobic mesophiles and psychrotrophic counts. The information obtained in the present study can be used to evaluate the potential use of propolis as product for sanitizing other vegetables and for developing other food preservation technologies, with impact on human health.

## 1. Introduction

Propolis is a material produced from the collected buds or resinous substances of plants by bees (*Apis melifera* L.). These substances are mixed with β-glycosidase enzyme of their saliva, partially digested and added to bee wax to form the final product. Propolis is used by the bees to defend the beehives from the invaders, causing death by asphyxia, and promotes conservation of their bodies, protecting the beehive from the resultant plagues of putrefaction. Another propolis function is the thermal isolation of the beehive, being used to fill eventual cracks or apertures.

The chemical composition of propolis is highly variable mainly due to the variability of plant species growing around the hive, together with other factors such as climatic conditions, soil type and beekeeper activities. In spite of the possible differences in composition, more than 300 different compounds have been identified in this natural product [1], sharing considerable similarity in their overall chemical nature: 50% resin, 30% wax, 10% essential oils, 5% pollen and 5% of other organic compounds [2].

Propolis has been used since the primordial times due to its therapeutic properties. In the last years, this product has been the subject of diverse studies and reviews [3,4], which gave scientific support to their biological and pharmacological properties such as antibacterial, antiviral, antioxidant [5], hepatoprotective [6], cariostatic [7] and anticancer [8].

Humans today have a nutritional environment that differs from that for which our genetic constitution was selected. Indeed, nowadays, the industrialized society’s diets are characterized, by a decrease in fruits, vegetables, complex carbohydrates and fibers. Nutritional organizations have been promoting consumption of fresh vegetables and fruits as part of a healthy diet to prevent disease. At the same time, the retail industry has developed value added products such as ready-to-eat (RTE) and minimally processed vegetables, which meet consumer demands for convenience, healthiness and variety.

However, the occurrence of outbreaks of foodborne illness associated with contaminated fresh vegetables is a serious public health problem with increasing prevalence, particularly in immunocompromised individuals [9,10].

There are many potential sources of products’ contamination, beginning at the pre-harvest phase and ending in the consumer’s kitchen. Critical control points remain important to avoid and/or reduce contamination. The practice of washing and sanitizing vegetables before consumption by the consumers has the potential to reduce the overall microflora of leafy vegetables. Different sanitization methods and several types of sanitizers have been used to reduce populations of pathogens on produce or prevent growth of pathogenic and spoilage microorganisms that may cause foodborne illnesses and/or loss of food quality [11,12]. Chlorinated compounds, mainly sodium hypochlorite are the most used. However, several reports have questioned its efficacy and given emphasis to the formation of trihalomethanes, which are carcinogenic compounds [13,14].

A promising alternative sanitization process could involve the use of propolis. Indeed, this product has attracted much attention in recent years as a useful or potential agent with application in the food industry for health foods, beverages and nutritional supplements. Moreover, it has also been tested as food preserver due to its bactericidal and bacteriostatic properties [15,16,17,18].

In this context, the present study aimed to determine: (a) the microbiological quality (mesophilic, psychrotrophic, fecal coliforms, *Escherichia coli*, sulphite reducing clostridium spores, *S. aureus* and *Salmonella*) of commercially available ready-to-eat and fresh whole head lettuces and (b) the efficacy of different washing solutions (tap water, sodium hypochlorite and propolis) and contact times on the microbial quality. The results obtained can be used to evaluate the potential use of propolis in the sanitization of other vegetables and in the development of new food preservation technologies.

## 2. Results and Discussion

### 2.1. Propolis Characterization

According to Sforcin *et al.* [18] a considerable part of papers dealing with different aspects of the biological properties of propolis are of limited usefulness, although they report “strong”, “remarkable” or “significant” activity. The reason is the lack of basis for comparison and scientific evaluation of the results, because they do not refer to the chemical nature of the studied propolis samples. These studies only report that the tests have been performed with extracts of propolis. However, it is important to note that propolis biological properties should be linked to a detailed investigation of its chemical composition and to its botanical sources [19]. For this reason, propolis used in the present study was characterized for: pollen grains spectra, physicochemical (moisture, ash, electrical conductivity, pH and wax) and bioactive compounds (total phenolics and flavonoids).

Even though the palynological analyses revealed the presence of 9 botanical families, only 5 were present on all samples, whose average percentages are presented on Table 1. Indeed, in some samples minor pollens belonging to 4 other botanical families were found, however, since the percentages were lower than 3.0% and the dispersion between samples was high, these are not presented.

The different botanical families found provide evidence for the classification of the product as heterofloral. The most dominant family present, with a percentage of 41.4% ± 3.8% was *Erica* sp., followed by *Populus* sp., *Echium* sp., *Castanea* sp., *Cytisus* sp. and *Quercus* sp. These results were already expected, since, in line with Pires *et al.* [20], the most relevant botanical families of the Northeast of Portugal are *Ericaceae*, *Boraginaceae*, *Fagaceaea* and *Salicaceae*. Considerable percentages of *Populus* sp. have also been reported by Falcão *et al.* [21] and Dias *et al.* [22], who analysed Portuguese propolis. Moreira *et al.* [5] obtained *Castanea sativa* as the dominant species in propolis collected from the same area but from different apiaries.

**Table 1:** Palynological spectrum of the propolis samples.

Family	Pollen Type	Frequency *	Range (%)	Mean (%)	SD
*Boraginaceae*	*Echium* sp*.*	SP	11.4–18.0	16.2	4.6
*Ericaceae*	*Erica* sp.	SP	36.7–43.9	41.4	3.8
*Fabaceae*	*Cytisus* sp.	IMP	3.7–6.6	5.2	1.3
*Fagaceae*	*Castanea* sp.	IMP	8.0–14.0	11.9	4.1
	*Quercus* sp.	MP	2.2–3.8	3	0.7
*Salicaceae*	*Populus* sp.	SP	22.0–25.5	22.3	2.9

* The following terms were used for frequency classes: SP, secondary pollen (16%–45%); IMP, important minor pollen (3%–15%) and MP, minor pollen (1%–3%).

Studies concerning the physicochemical characteristics of propolis have often focused on the presence of polyphenols and flavonoids [5,21,23,24]. More recently, however, parameters like moisture, insoluble and soluble substances, pH, conductivity, ash and waxes have received attention [22]. The values obtained in the present study for the physicochemical parameters are presented in Table 2. These results are corroborated by the values obtained by Dias *et al.* [22], who studied propolis samples from four different cities of Portugal. Sousa *et al.* [25] obtained similar values for moisture and ash, even though great differences were found among the Brazilian samples studied. Concerning the waxes and the soluble solids, the first were higher and the second were much lower than those obtained in the present study. These differences are due to different botanical and geographical origins. Indeed, the composition of propolis is highly variable due to the diversity of plants around the hive from which the bees collect the exudates [1].

**Table 2:** Physicochemical characteristics of the propolis samples.

Result	Moisture	Soluble Substances	Insoluble Substances	pH	Conductivity (mS/cm)	Ash (%)	Waxes (%)	Phenolic Compounds (GAEs) ^a^	Flavonoid Compounds (CAEs) ^b^
Mean	6.2	65.3	38.4	4.8	2.1	2.5	10.7	29.5	10.3
SD	0.5	3.7	2.5	0.1	0.3	0.1	1.9	4.2	2.5
Range	5.8–6.6	60.2–67.3	36.4–40.0	4.7–4.9	2.0–2.5	2.4–2.5	9.9–11.6	23.3–32.0	8.9–11.9

^a^ Total phenols content were expressed as mg of galic acid equivalents per g of propolis (GAEs); ^b^ Total flavonoids content were expressed as mg of catechin equivalents per g of propolis (CAEs).

### 2.2. Effectiveness of Sanitizing Treatments

To determine the microbiological quality of the raw vegetable, a lettuce sample was analyzed at the beginning, unwashed, to evaluate the initial microbiological load. As expected, RTE lettuce showed minor contents of bacterial burden compared to that of fresh whole head (FWH) lettuce (Table 3). However, results show that both the RTE and FWH lettuce analyzed presented borderline microbiological quality according to the Portuguese guidelines for RTE-salads (CFU/g): aerobic mesophilic count, unsatisfactory ˃10^6^ and satisfactory ≤10^4^.

**Table 3:** Results (log_10_ CFU/g) of the microbiological analysis made to the ready-to-eat (RTE) and fresh whole head (FWH) lettuce submitted to different sanitization processes.

Treatment *	Aerobic Mesophiles		Psychrotrophic		Fecal Coliforms		*E. coli* ^a^		*Salmonella* ^b^		*S. aureus* ^a^		Sulphite-Reducing Clostridia ^c^	
	RTE	FWH	RTE	FWH	RTE	FWH	RTE	FWH	RTE	FWH	RTE	FWH	RTE	FWH
–	4.95	5.8	5.25	5.8	1.9	2.15	ND	ND	ND	D	<2	˂2	ND	ND
TW-15'	3.85	4.7	4.6	4.7	1.4	1.95	ND	ND	ND	D	˂2	˂2	ND	ND
PS-15'	2.4	3.2	3.1	3.4	0.5	1.2	ND	ND	ND	ND	˂2	˂2	ND	ND
SH-15'	2.45	3.35	3.5	3.8	0.8	1.4	ND	ND	ND	ND	˂2	˂2	ND	ND
TW-30'	3.6	4.45	3.45	4.7	1.4	0.9	ND	ND	ND	ND	˂2	˂2	ND	ND
PS-30'	2.15	2.55	2.25	3	0.3	1.1	ND	ND	ND	ND	˂2	˂2	ND	ND
SH-30'	2.25	2.75	2.6	3.25	0.6	1.3	ND	ND	ND	ND	˂2	˂2	ND	ND

* Tap water (TW), sodium hypochlorite (SH) and propolis solution (PS) for contact time of 15 and 30 min (TW-15', TW-30', SH-15', SH-30', PS-15', PS-30'). ^a^ in 1 g; ^b^ in 25 g; ^c^ in 0.01 g.

Recent studies revealed that ready-to-eat leafy vegetable had poor microbiological quality and it indicates the need of adoption of hygienic practices by food processors and consumers to minimize the risks of transmission of foodborne pathogens through this kind of food [26,27,28].

Mesophilic aerobic and psychrotrophic counts are equal in FWH lettuce. However, the number of psychrophilic bacteria present in RTE sample (5.25 log_10_ CFU/g) was higher than the number of mesophilic bacteria (4.95 log_10_ CFU/g). The refrigeration temperature used in the storage of RTE vegetables extended the shelf life of these products, slowing down the microorganism growth rate, but it is selective for psychrotrophic microorganisms.

*E. coli* and sulphite-reducing clostridia were not detected in both RTE and FWH samples. However, *Salmonella* was detected in FWH. In fact, contaminated manure and irrigation water play important roles in contaminating vegetables with *Salmonella* [29].

The effects of sodium hypochlorite (SH) and propolis solution (PS), as well as the control treatment (immersion in tap water, TW) on microbiological counts present on both RTE and FWH lettuce are also shown in Table 3 and Table 4.

**Table 4:** Results of the mean reduction (log_10_ CFU/g) on the microbiological analysis made to the ready-to-eat (RTE) and fresh whole head (FWH) lettuce submitted to different sanitization processes.

Treatment *	Aerobic Mesophiles		Psychrotrophic		Fecal Coliforms	
	RTE	FWH	RTE	FWH	RTE	FWH
TW-15'	1.1	1.1	0.65	1.1	0.5	0.2
PS-15'	2.55	2.6	2.15	2.4	1.4	0.95
SH-15'	2.5	2.45	2.15	2	1.1	0.75
TW-30'	1.35	1.35	1.8	1.1	0.5	1.25
PS-30'	2.8	3.25	3	2.8	1.6	1.05
SH-30'	2.7	3.05	2.65	2.55	1.3	0.85

* Tap water (TW), sodium hypochlorite (SH) and propolis solution (PS) for contact time of 15 and 30 min (TW-15', TW-30', SH-15', SH-30', PS-15', PS-30').

The control treatment with TW applied to FWH lettuce for contact time of 15 and 30 min. was not sufficient to reduce the microorganism population to acceptable levels (≤4 log_10_ CFU/g) [30]. However, in relation to RTE lettuce, TW immersion was sufficient to reduce aerobic mesophiles under values minor than 4 log_10_ CFU/g.

In relation to mean reduction on aerobic mesophiles, psychrotrophic and fecal coliforms, involving RTE and FWH lettuce, the SH and PS treatments showed the same pattern of variation (Table 4). In all cases, PS was slightly more effective in microbiological reduction in comparison with commercial SH. Reductions between two and three log cycles were obtained with PS on aerobic mesophiles and psychrotrophic counts at contact times of 15 and 30 min. As described previously, the variability of the results was assessed with the Kruskal-Wallis test (Table 5), followed by the Mann-Whitney post hoc test, with a significance level of 5%. It was verified that the three types of treatment significantly influenced the microbiological parameters, both for unprocessed and minimally processed lettuce. Regarding the time of treatment, significant differences were not found, with the exception of the results obtained for the psychrotrophic microorganisms of ready-to-eat lettuce.

**Table 5:** Results of the Kruskal–Wallis test regarding the different times and types of treatment applied to the fresh whole head (FWH) and ready-to-eat (RTE) lettuce. *p* = 0.05.

Microorganism	FWH *p*-Value		RTE *p*-Value	
	Treatment	Time	Treatment	Time
Aerobic mesophiles	<0.001	0.047	<0.001	0.046
Psychrotrophic	<0.001	0.094	0.008	0.004
Fecal coliforms	<0.001	0.533	<0.001	0.710

It is worth mentioning that SH is one of the most important types of chlorine-releasing agent (CRAs) compounds used for both antiseptic and disinfectant purposes. Excellent reviews that deal with the chemical, physical and microbiological properties of CRAs are available [31]. In H_2_O, SH ionizes to produce Na^+^ and the hypochlorite ion, OCl^−^, which establishes an equilibrium with hypochlorous acid, HOCl. Between pH 4 and 7, chlorine exists predominantly as HClO, the active moiety, whereas above pH 9, OCl^−^ predominates. Surprisingly, despite being widely studied, the actual mechanism of action of CRAs is not fully known.

Importantly, certain microorganisms, have innate chlorine resistance and may also develop acquired resistance following exposure to chlorine [12]. Some studies suggest a relationship between microbial resistance to sanitizers and microbial resistance to antibiotics used therapeutically [12]. Moreover, it is now well-recognized that chlorine may incompletely oxidize organic materials to produce undesirable byproducts, such as chloroform or other trihalomethanes, that have that have been linked to cancers, miscarriages and birth defects [32].

In the other hand, propolis is one of the most potent natural antibiotics, safe for human health and does not induce germ resistance. It seems that rather the sum of the propolis antimicrobial components than individual substances are responsible for the antimicrobial action [33]. Even though the action mechanisms are not fully understood, the antimicrobial activity is potentially due to phenolic and flavonoids. These compounds increase the permeability of the inner bacterial membrane, nullifying its potential, decreasing ATP production, membrane transport and its mobility [34]. In addition, they inhibit DNA gyrase, involved in the mechanism of DNA and RNA synthesis of bacteria [35].

## 3. Experimental Section

### 3.1. Chemicals and Reagents

3,4,5-Trihydroxybenzoic acid (gallic acid; GA), (2*R*,3*S*)-2-(3,4-dihydroxyphenyl)-3,4-dihydro-2*H*-chromene-3,5,7-triol [(+)-catechin; CA] and ethanol were obtained from Sigma Chemical Co. (St. Louis, MO, USA). The Folin–Ciocalteu reagent (FCR) and sodium carbonate (Na_2_CO_3_) were obtained from Merck (Darmstadt, Germany). H_2_SO_4_, KOH, aluminium chloride (AlCl_3_), NaNO_2_ and NaOH were purchased from Acros Organic (Geel, Belgium). Methanol (MeOH) was obtained from Pronolab (Lisboa, Portugal). Amukina^®^ was purchased in supermarket. High purity water (18 MΩ cm), which was used in all experiments, was obtained from a Milli-Q purification system (Millipore, Bedford, MA, USA). All growth media were purchased from Oxoid Ltd. (Himedia, Telangana, India).

### 3.2. Apparatus

Spectrophotometric measurements were made using a Unicam Helios Alpha UV-visible spectrometer (Thermo Spectronic, Cambridge, UK). Evaporation of organic solvents was performed with a rotavapor system, consisting of a rotary vacuum evaporator (Heidolph VV. 2000, Leuven, Belgium) with a water bath and a B169 vacuum pump (Buchi, Flawil, Switzerland). The examination of the pollen slides was carried out with a Leitz Diaplan microscope (Leitz Messtechnik GmbH, Wetzlar, Germany). Stomacher Lab-Blender (Seward type 400, London, UK) and stainless steel metal sieves with a pan collector were supplied by Filtra (Filtra Vibracion S.L., Badalona, Catalunya). An electric laboratory furnace SNOL 8.2/1100-1 (AB ‘‘Umega’’, Utena, Lithuania) was used to determine ash content.

### 3.3. Propolis

#### 3.3.1. Sampling

Propolis samples (*n* = 37) were collected by beekeepers in the fall of 2012 from *Apis mellifera* hives located in Bragança, Portugal (41°48'N; 06°45'W). Samples were obtained after honey extraction by scratching the hive walls and frames. Upon receipt, each sample was inspected in order to find rests of bees, wood, plant, pupa of moth, among others. The major visible impurities were removed from the samples. Samples were weighed (980 g) and frozen at −20 °C until analysis.

After the palynological identification, 10 propolis samples with the most identical botanical origin were selected from the initial 37 samples, in order to have a composite sample, consisting of a mixture of several individual samples. This method creates a more representative sample of the characteristics of propolis of the region under study, which was used for the sanitization of lettuce.

#### 3.3.2. Palynological Identification

Palynological processing of the samples followed the standard methodology, described in detail previously [5]. In brief, 0.5 g of scraped propolis was extracted overnight with ethanol. Next, the sediment was treated with KOH (10%), sonicated for 15 min and sieved through a 20 mesh stainless steel screen to eliminate large fragments. In this stage, three propolis microscope slides were mounted with sediment obtained after centrifugation (10,000× *g* for 1 min) for observation of plant trichomes and other organic residues that may be destroyed in sequence. Then acetolysis was applied, and two additional microscope slides were prepared using glycerin jelly, one stained with basic fuchsine and the other without stain. Pollen grain identification was performed by optical microscope with total magnification (400× and 1000×). A reference collection of CIMO—Mountain Research Centre (Agricultural College of Bragança) and different pollen morphology guides were used for the recognition of the pollen types [36]. The following terms were used for frequency classes: predominant pollen (PP, more than 45% of pollen grains counted), secondary pollen (SP, 16%–45%), important minor pollen (IMP, 3%–15%) and minor pollen (MP, 1%–3%).

#### 3.3.3. Physicochemical Analysis

Analyses of the physicochemical properties of propolis samples were reported previously in detail [22]. The evaluated parameters were: moisture (%), ash (%), electrical conductivity (mS/cm), pH, soluble substances, insoluble substances and wax (%).

#### Moisture

Five grams of propolis were dried in a mechanical convection oven at 105 °C for 1 h. After this time, it was removed and allowed to cool to room temperature and weighted again. The procedure was repeated to stabilize the weight. The water content was determined using the Equation (1), in which A1 = weight of sample; A2 = weight of dried sample:

Moisture (%) = 100 × (A1 − A2)/A1
(1)

#### Ash Content

The method used in the experiments to determine the mineral content and other inorganic matter in propolis consisted of the desiccation of an amount of 5 g, for each propolis sample, in a platinum dish. To do so, they were kept in the thermostat at 80 °C for 4 h, after which the samples underwent calcination at 550 °C to constant mass. Total ash contents, expressed as the percentage of residue left after dry oxidation by weight (%), was calculated from the Equation (2), where m_1_ is the mass of dish and ash, m_2_ the mass of platinum dish prior to calcination and m_0_ is the mass of the propolis taken:

Ash (%) = (m_1_ − m_2_/m_0_) × 100 (2)

#### Electrical Conductivity

Electrical conductivity of a propolis solution at 20% (*w*/*v*) (dry matter basis) in methanol was measured at 20 °C in a conductimeter.

#### pH

Propolis pH was measured in a solution prepared with 10 g of propolis in 75 mL of methanol.

#### Soluble and Insoluble Substances

To one gram of each propolis sample, 250 mL of ethanol was added. The mixture was shacked in an automatic mixing machine and after 30 min the solution was filtered and the insoluble solids weighed. The soluble solids (SS) were determined by the difference between sample weight (SW) and insoluble weight (IW). The result was expressed in percentage, by applying the Equation (3) for soluble solids:

SS (%) = (SW − IW/SW) × 100
(3)

Equation (4) is for insoluble substances:

IS (%) = (IW/SW) × 100
(4)

#### Wax

Wax was weighed, and 250 g of each sample was added to 750 mL of methanol. The mixture was placed in a freezer (−20 °C) overnight. Afterwards, the solution was filtered to obtain the wax. The wax was expressed in percentage W (%) using the sample weight (SW) and the wax weight (WW). The Equation (5) used was:

W (%) = (WW/SW) × 100
(5)

#### 3.3.4. Extract Preparation

For the preparation of propolis extracts (PE), ultrasound-assisted solvent extraction (USE) was employed. In brief, powdered propolis and MeOH were mixed (1:2) (*w*/*v*) in a 100-mL flask, sonicated for 60 min. and then centrifuged at 5000 rpm for 5 min. Next, the supernatant was taken out and the solid residue was extracted again and supernatants combined. MeOH of the extraction solution was evaporated in a vacuum evaporator. Finally, the dried PE was frozen at −20 °C until analysis.

#### 3.3.5. Total Phenolics and Flavonoids

Total phenolic contents in the extracts were recorded using the Folin–Ciocalteu method as described previously [5]. Briefly, a dilute solution of each PE in MeOH (MeOH–propolis; 500 µL of 1:10 g/mL) was mixed with 500 µL of Folin-Ciocalteu reagent (FCR) and 500 µL of Na_2_CO_3_ (10% *w*/*v*). After incubation in the dark at room temperature for 1 h, the absorbance of the reaction mixture at 700 nm was determined against the blank (the same mixture without the MeOH–propolis). GA standard solutions were used for constructing the calibration curve (*y* = 0.3882*x* + 0.048; *R*^2^ = 0.9992). Total phenol contents were expressed as mg of GA equivalents per g of propolis (GAEs).

For flavonoid contents, the aluminium chloride method was used [5]. In brief, MeOH–propolis (250 µL) was mixed with 1.25 mL of distilled H_2_O and 75 µL of a 5% NaNO_2_ solution. After 5 min, 150 µL of a 10% AlCl_3_–H_2_O solution was added. After 6 min, 500 µL of 1 M NaOH and 275 µL of distilled H_2_O were added to the mixture and vortexed. The solution was well mixed and the intensity of pink color was measured at 510 nm. CA standard solutions were used for constructing the calibration curve (*y* = 20.47*x* − 0.024; *R*^2^ = 0.9996). Total flavonoid contents were expressed as mg of CA equivalents per g of propolis (CAEs).

### 3.4. Lettuce

#### 3.4.1. Sampling

Commercially available ready-to-eat (RTE, *n* = 6) and fresh whole head (FWH, *n* = 6) lettuces were obtained in different supermarkets in Bragança (Portugal). The samples were collected in the state in which they were available for consumer purchase, placed inside sterile sample bags with ice packs, and delivered to the laboratory for analyses within 1 h after collection. Samples were kept stored for a maximum of 3 h before use in experiments.

#### 3.4.2. Preparation of Sanitizing Solutions

Two sanitizing solutions were employed: sodium hypochlorite (SH) and propolis solution (PS). Tap water (TW) was used as a control. SH used in a commercial available presentation called Amukina^®^ (11.5 mg/mL), was prepared according to the manufacture’s specifications by adding 50 mL of Amukina^®^ for every 2.5 L of H_2_O. PS was prepared at 2% by dissolving 40 mg of the PE in 100 mL of H_2_O. SH and PS solutions were used immediately after preparation.

#### 3.4.3. Sanitizing Treatments

In FWH lettuces the outer leaves were removed depending on the visual quality before processing. RTE and FWH were cut into small pieces (5 × 10 cm) using a sharp knife. These freshcut samples were washed carefully with gentle agitation in separate buckets each containing TW, SH and PS for contact time of 15 and 30 min (TW-15', TW-30', SH-15', SH-30', PS-15', PS-30') individually and separately. After the treatment, the samples were washed with water and dewatered for 1 min with a manual centrifugal dryer.

### 3.5. Microbiological Analysis

The following microorganisms were investigated: mesophilic, psychrotrophic, fecal coliforms, *Escherichia coli*, sulphite reducing clostridium spores, *S. aureus* and *Salmonella*.

#### 3.5.1. Sample Preparation

Prior to the sanitizing treatments, 10 g of each RTE and FWH samples were aseptically taken and homogenized using a pre-sterilized stomacher for 3 min with 90 mL of pre-chilled (4 ± 0.5 °C) sterile peptone-physiological saline solution (0.1% neutral peptone +0.85% NaCl in sterile deionized H_2_O, pH = 7.0 ± 0.05). Decimal serial dilutions were prepared from this homogenate in the same chilled sterile diluents (1:10 (*w*/*w*)).

#### 3.5.2. Enumeration of the Mesophilic and Psychrotrophic Microorganisms

The aerobic mesophilic and psychrotrophic microorganisms were counted by incorporation of 1 mL of each dilution into standard Plate Count Agar, and incubated aerobically at 30 °C for 72 h and 7 °C for 10 days for the enumeration of mesophilic and psychrotrophic bacteria, respectively as recommended [37]. Microbial counts were expressed in log colony-forming units per gram (log CFU/g).

#### 3.5.3. Enumeration of Total Coliforms and *Escherichia coli* (*E.coli*)

Enumeration of coliforms and *E. coli* was done using the SimPlate CEc-CI method [38] with multiple test medium (BioControl System, Bellevue, WA, USA), according to the manufacturer’s instructions and previously procedures of [39]. One milliliter from the basic dilution was placed in the center of the SimPlate plating device, and 9 mL of a mixed nutrient agar with blue color was added at the same spot. The SimPlate was rotated in order to disperse the sample and remove air-bubbles. The SimPlates were stacked and stored at 37 ± 1 °C for 24–28 h. Wells were counted positive for total coliforms on the basis of the color change and counted positive for *E. coli* on the basis of color change and fluorescence under UV light. The coliform and *E. coli* populations were determined on the basis of the number of positive wells correlated with the SimPlate conversion table, which generated a most probable number (MPN) per gram of sample.

#### 3.5.4. Enumeration of Sulphite Reducing Clostridium Spores

For Sulphite-reducing clostridia counting, aliquots of 10, 5, 1 and 0.1 mL of the initial suspension were added to an empty tube, thermally treated at 80 °C for 15 min and covered with Differential Reinforced Clostridial Broth, and incubated at 37 °C for 5 days. At the end, the black colonies were counted. The results are expressed as presence of Sulphite-reducing clostridia in 0.01 g [40].

#### 3.5.5. Enumeration of *S. aureus*

The detection was performed according to [41]. Following the existent legislation, serial dilutions of the sample were inoculated in Baird–Parker Broth with Egg Yolk Tellurite and Sulfadimidine Solution for 24 h at 37 °C. Afterwards, 3–5 characteristic colonies were selected in order to verify the presence of coagulase and catalase. Microbial counts were expressed in log colony-forming units per gram (log CFU/g).

#### 3.5.6. Detection of *Salmonella* sp.

The detection of *Salmonella* sp. in the samples was carried out using the immunodifusion 1–2 test [38]. Results are obtained 16–20 h after pre-enrichment in buffered peptone water and interpreted by observing the development of an immunoband, a characteristic immobilization pattern of cells.

### 3.6. Statistical Analysis

Each sample was analysed in triplicate. Regarding the statistical analysis, two well-known tests of normality were used: the Kolmogorov–Smirnov Test and the Shapiro–Wilk Test. The Levene’s test was also used in order to assess variance homogeneity. Indeed, the assessment of normality and variance homogeneity of data are prerequisites for many statistical tests, since these are underlying assumptions in parametric testing. Even though the results obtained for some parameters had normal distribution, the variance homogeneity was not verified in any of the cases. In this context, for each parameter, the differences between the samples were analysed using the Kruskal–Wallis test, the non-parametric analogue of a one-way ANOVA, with a level of significance of 5%. Mann–Whitney test was used as a *post-hoc* test. This treatment was carried out using SPSS version 21 (IBM Software, Toronto, ON, Canada).

## 4. Conclusions

This study is the first attempt to evaluate the effect of propolis as an alternative to sodium hypocholorite in the sanitization of lettuce. Both treatments showed similar effectiveness in reducing the microbial load. Overall, these findings support that propolis is a promising sanitizer agent. In relation to mean reduction on aerobic mesophiles, psychrotrophic and fecal coliforms, in all cases, PS was slightly more effective in microbiological reduction in comparison with commercial SH. Reductions between two and three log cycles were obtained with PS on aerobic mesophiles and psychrotrophic counts at contact times of 15 and 30 min. In future studies, tests will be performed using different vegetables, controlled bacterial load and organic matter in order to determine the effectiveness of propolis, and take advantage of this natural product, which is apparently free of any non-desirable secondary effects.
